# Regulation of the apoptosis/autophagy switch by propionic acid in ventromedial hypothalamus of rats with type 2 diabetes mellitus

**DOI:** 10.1016/j.heliyon.2022.e11529

**Published:** 2022-11-14

**Authors:** Larysa Natrus, Yuliia Osadchuk, Olha Lisakovska, Toralf Roch, Nina Babel, Yuliia Klys, Dmytro Labudzynskyi, Yuri Chaikovsky

**Affiliations:** a Department of Modern Technologies of Medical Diagnostics & Treatment, Bogomolets National Medical University, 34 Peremoha Avenue, Kyiv 03115, Ukraine; b Department of Histology and Embryology, Bogomolets National Medical University, 34 Peremoha Avenue, Kyiv 03115, Ukraine; c Department of Biochemistry of Vitamins and Coenzymes, Palladin Institute of Biochemistry, 9 Leontovicha Str., Kyiv 01054, Ukraine; d Center for Translational Medicine and Immune Diagnostics Laboratory, Medical Department I, Marien Hospital Herne, University Hospital of the Ruhr University Bochum, Hölkeskampring 40, 44625 Herne, Germany; e Charité – Universitätsmedizin Berlin, Corporate Member of Freie Universität Berlin, Humboldt-Universität zu Berlin, Berlin Center for Advanced Therapies (BeCAT), Augustenburger Platz 1, 13353 Berlin, Germany

**Keywords:** Propionic acid, Metformin, Type 2 diabetes, Ventromedial hypothalamus, Apoptosis, Autophagy

## Abstract

**Background:**

Hypothalamic dysregulation may cause abnormal glucose metabolism and type 2 diabetes mellitus (T2DM). The balance between autophagy and apoptosis is important for maintaining cellular/tissue homeostasis and may be disrupted in T2DM.

**Objectives:**

Since propionic acid (PA) exerts neuroprotective effects, the aim was to investigate its effects on apoptosis/autophagy switch in the ventromedial hypothalamus (VMH) of T2DM rats.

**Materials and methods:**

Male Wistar rats were divided: 1) control; 2) T2DM; groups that received (14 days, orally): 3) metformin (60 mg/kg); 4) sodium salt of PA (60 mg/kg); 5) PA + metformin. Western blotting (Bax, Bcl-xl, LC3, Beclin-1, caspase-3), RT-PCR (Bax, Bcl-xl, LC3, Beclin-1), transmission electron microscopy and immunohistochemical staining (Bax, Bcl-xl) were performed on the VMH samples.

**Results:**

T2DM-induced apoptosis and mitoptosis, enlarged endoplasmic reticulum (ER) tubules/cisterns were observed in VMH, and accompanied by an imbalance of pro- and anti-apoptotic factors: elevation of pro-apoptotic markers Bax and caspase-3, decrease in autophagy protein LC3 and anti-apoptotic Bcl-xl. Metformin and PA administration partially improved VMH ultrastructural changes by reducing mitochondrial swelling and diminishing the number of apoptotic neurons. Metformin inhibited neuronal apoptosis, however, caused reactive astrogliosis and accumulation of lipofuscin granules. Elevated number of autophagosomes was associated with the LC3, Beclin-1 and Bcl-xl increase and decrease in Bax and caspase-3 vs. T2DM. PA switched cell fate from apoptosis to autophagy by elevating LC3 and Beclin-1 levels, increasing Bcl-xl content that altogether may represent adaptive response to T2DM-induced apoptosis. PA + metformin administration lowered relative area of ER membranes/cisterns vs. control, T2DM and metformin, and was optimal considering ratio between the pro-apoptotic, anti-apoptotic and autophagy markers.

**Conclusion:**

T2DM was associated with apoptosis activation leading to impairments in VMH. PA in combination with metformin may be effective against diabetes-induced cell death by switching apoptosis to autophagy in VMH.

## Graphical abstract

**Figure:**
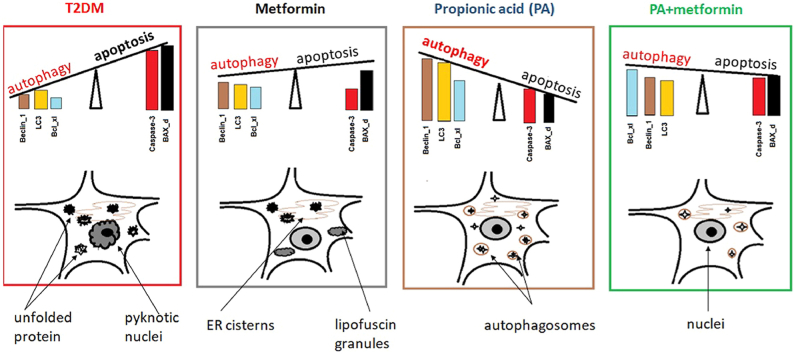

## 1 Introduction

Type 2 diabetes mellitus (T2DM) is the most common chronic metabolic disorder, the clinical importance and global pandemic character of which is confirmed by the World Health Organization (WHO) [1]. One of the frequently occurring complications of diabetes is the damage to the peripheral and autonomic nervous systems, that is diagnosed in up to half of all patients with T2DM [2]. Globally, obesity, lifestyle factors, genetic predispositions, gut dysbiosis, and mitochondrial deregulation were shown to be among the major risk factors for the T2DM-associated neuropathy [3]. Diabetes is also associated with gradually developing injury of the central nervous system (CNS), including hypothalamus [4]. The neurons of the ventromedial (VMH), lateral (LHA) and arcuate (ARK) nuclei of the hypothalamus are responsible for the regulation of the energy balance of the organism. T2DM has a disrupting effect on the tight balance the molecular pathways and cellular processes in the hypothalamic zones, that can be one of the fundamental reasons of impaired glucose metabolism, and may be accompanied by the neuroinflammation and impaired neurogenesis in general [5, 6, 7]. Thus, a bidirectional link may be suggested between T2DM development and hypothalamic dysfunction and both may be considered as reasons and consequences for each other.

Existing evidence suggests that an excess of glucose and fatty acids disrupts endoplasmic reticulum (ER) functioning and causes the cell damage [8]. Chronically stressed ER may induce the impaired proinsulin synthesis, folding, and protein processing, generate pro-apoptotic signals, and initiate mitochondrial apoptosis [9]. Thus, the cell fate upon ER stress is determined by the balance of cell survival and cell death molecular triggers, that mainly regulated by the unfolded protein response (UPR) signaling. ER stress-induced autophagy is required for cell survival, likely through the compensatory removal of disorganized ER (which results from the UPR activation), along with the misfolded proteins, that altogether represent the ER-associated degradation (ERAD) mechanism [10]. ER stress-associated mechanisms may influence the autophagy process through the involvement of PERK (protein kinase R-like ER kinase)-mediated signaling pathways [11, 12]. Our previous results showed that the elevated level of PERK, one of the major UPR sensors, in VMH under diabetic condition was essential for an autophagy induction [13]. Thus, particular attention should be paid in order to explore how the regulation of switch between the processes of apoptosis and autophagy as a tightly coordinated system for maintaining intracellular homeostasis and an important factor in the development of T2DM-associated hypothalamic dysfunction occurs.

Autophagy is a fundamental cellular homeostasis program, that under normal physiological condition is responsible for the utilization of the damaged organelles and misfolded proteins by delivering them to lysosomes for degradation [14, 15, 16]. However, in pathological context, including the development of diabetes, autophagy also plays a pivotal role [17]. Moreover, autophagic activity is important for maintenance of neuronal functioning since these cells are unable to dispose the aggregated proteins [18, 19]. ER stress, associated with chronic neurodegenerative disorders often stimulates autophagic activity, however, failure in clearing the aggregated proteins and the impairment of UPR lead to the accumulation of misfolded proteins and the progression of neurodegeneration [20, 21]. The association between ER stress, UPR system state, cell death and neuropathology is partially established, however, the role of this interplay upon T2DM-associated hypothalamic dysfunction should be studied in detail.

The commonly used autophagy markers are Beclin-1 and microtubule-associated protein 1 light chain 3 (LC3). Moreover, Beclin-1 also plays an important role in T2DM progression. It was shown that Beclin-1 is involved in the pathogenesis of diabetic nephropathy [22]. Related to brain, it was shown that Beclin-1-mediated autophagy in hippocampus probably plays an important role in cognitive and affective disorders of streptozotocin (STZ)-induced aged diabetic mice [23]. LC3 is frequently used as an additional autophagic marker in order to measure autophagic flux. LC3 is an ubiquitin-like protein that is associated with the autophagosomes during the autophagic process [24]. The ubiquitin-like conjugation of phosphatidylethanolamine (PE) to LC3 couples with the translocation of LC3 from the soluble fraction to autophagic membranes. Therefore, the localization of LC3-PE in autophagic membranes is a reliable marker of autophagy [25]. Thus, it is important to study the involvement of LC3 and Beclin-1 in the hypothalamic dysfunction under T2DM since it was shown that autophagy may mediate the protective effect of metformin on hyperglycemia-induced apoptosis of cardiomyocytes [26].

Other key cell death markers are proteins Bax and Bcl-xl, the members of the bcl-2 family, a group of apoptosis-mediating factors [27]. Bax is considered apoptosis-promoting, while Bcl-xl has apoptosis-inhibiting properties, therefore, their ratio characterizes the apoptotic process, a highly regulated form of programmed cell death. It is believed, that one of the main causes of diabetes-induced CNS alterations and peripheral neuropathy may be neuronal death, however, the way of neuronal death in VMH during T2DM remains unclear, and the question arises what is predominated type of cell death, apoptosis or autophagy.

Pharmacological modulation of the UPR signaling and regulation of the interplay between apoptosis and autophagy in disease-affected tissues may contribute to the prevention and/or treatment of neurodegeneration. It is of great significance to explore the new effective treatment options with the focus on apoptosis/autophagy switch modulation. First, in this study we used metformin, the most used anti-hyperglycemic agent. When initiating pharmacological treatment for T2DM, metformin is considered the first-line treatment for the anti-diabetic monotherapy due to its anti-hyperglycemic, anti-inflammatory, anti-apoptotic and anti-oxidative properties [28, 29]. Moreover, it is interesting to study the effect of metformin on apoptosis and autophagy in the light of the available data regarding its controversial influence on these processes on different cell and animal models. Recent study demonstrated the ability of metformin to trigger an autophagy by the AMP-activated protein kinase (AMPK) activation mechanism with the subsequent inhibition of mammalian target of rapamycin (mTOR), that is one of major inhibitor of the autophagic flux, as it was shown on human gastric adenocarcinoma cells [30].

To improve the clinical outcomes, an addition of a second oral supportive therapy in line with the metformin monotherapy is one option, which may include different antidiabetic compounds. However, the most modern approach for the treatment of neuropathy in diabetes mellitus includes pharmacological agents correcting the functional state of the “intestinal microflora – central nervous system” axis (gut-brain axis, GBA), with a special emphasis on the mitochondrial apparatus and the energy metabolism in neurons. In animal models, enteric-soluble short-chain fatty acids (SCFAs), among which propionate (propionic acid, PA) and acetate the most abundant, and other intestinal metabolites have been shown to normalize fasting blood glucose, insulin tolerance, and generally exert therapeutic effects in obesity [31]. In addition, PA administration reduces ER stress in bovine mammary epithelial cell culture [32]. However, data inconsistency regarding the action of PA in the neurotoxic context have been observed when reviewing the available experimental studies [33], therefore this issue probably needs to be considered in general A recent study indicated that autophagic flux is impaired in PA-treated hippocampal neurons. At the molecular level, the mitogen-activated protein kinase (MAPK)/extracellular signal-regulated kinase (ERK) pathway was activated and autophagic activity was impaired [33]. In addition, SCFAs act as energy substances to protect intestinal barrier and inhibit autophagy. However, no data are available related to the influence of PA on apoptosis/autophagy balance under T2DM condition.

Considering abovementioned, the aim of this study was to explore the regulatory effect of PA and metformin administration on the molecular mechanisms of switch between autophagy and apoptosis in the ventromedial nucleus of the hypothalamus in rats with the experimentally induced T2DM.

## 2 Materials and methods

### 2.1 Animals

The study was performed on male Wistar rats (176.8 ± 8.3 g) of 2–6 months of age. All rats were kept in a humidity-controlled house (24 ± 2 °C), 65 ± 5% humidity with 12 h dark/light cycling, and were allowed to drink and eat freely the standard, balanced rodent diet. All experimental procedures with animals were carried out in an accordance with national guidelines and international laws concerning an animal welfare: “European Convention for the protection of vertebrate animals used for experimental and other scientific purposes” (Strasbourg, 1986), “Bioethical expertise of preclinical and other scientific research conducted on animals” No. 3447-IV (Kyiv, 2006). All procedures were approved by the Bioethics Committee of the Bogomolets National Medical University (Protocol No 123 from 23/12/2019).

### 2.2 Experimental design

Each experimental group consisted of 12 animals, previously acclimated for one week: 1) the untreated control group; 2) the group with experimentally induced T2DM by high-fat diet (HFD) treatment for 3 months and additionally by a single injection of streptozotocin (STZ, 25 mg/kg of b.w., Sigma, USA) followed by a placebo treatment with neutral saline solution, that was orally administered for 14 days*;* 3) the T2DM group that received anti-hyperglycemic agent metformin (GLUKOFAGE, Merck Sante, France) dissolved in water for injection at a dose 60 mg/kg of b.w., for 14 days, orally on the background of T2DM; 4) the T2DM group that received sodium salt of propionic acid (PROPICUM®, Flexopharm Brain GmbH & Co, Germany) dissolved in water for injection at a dose 60 mg/kg of b.w., for 14 days, orally on the background of T2DM; 5) the T2DM group that received metformin (60 mg/kg of b.w., for 14 days, orally) and sodium salt of PA (60 mg/kg of b.w., for 14 days, orally).

**Figure 1:**
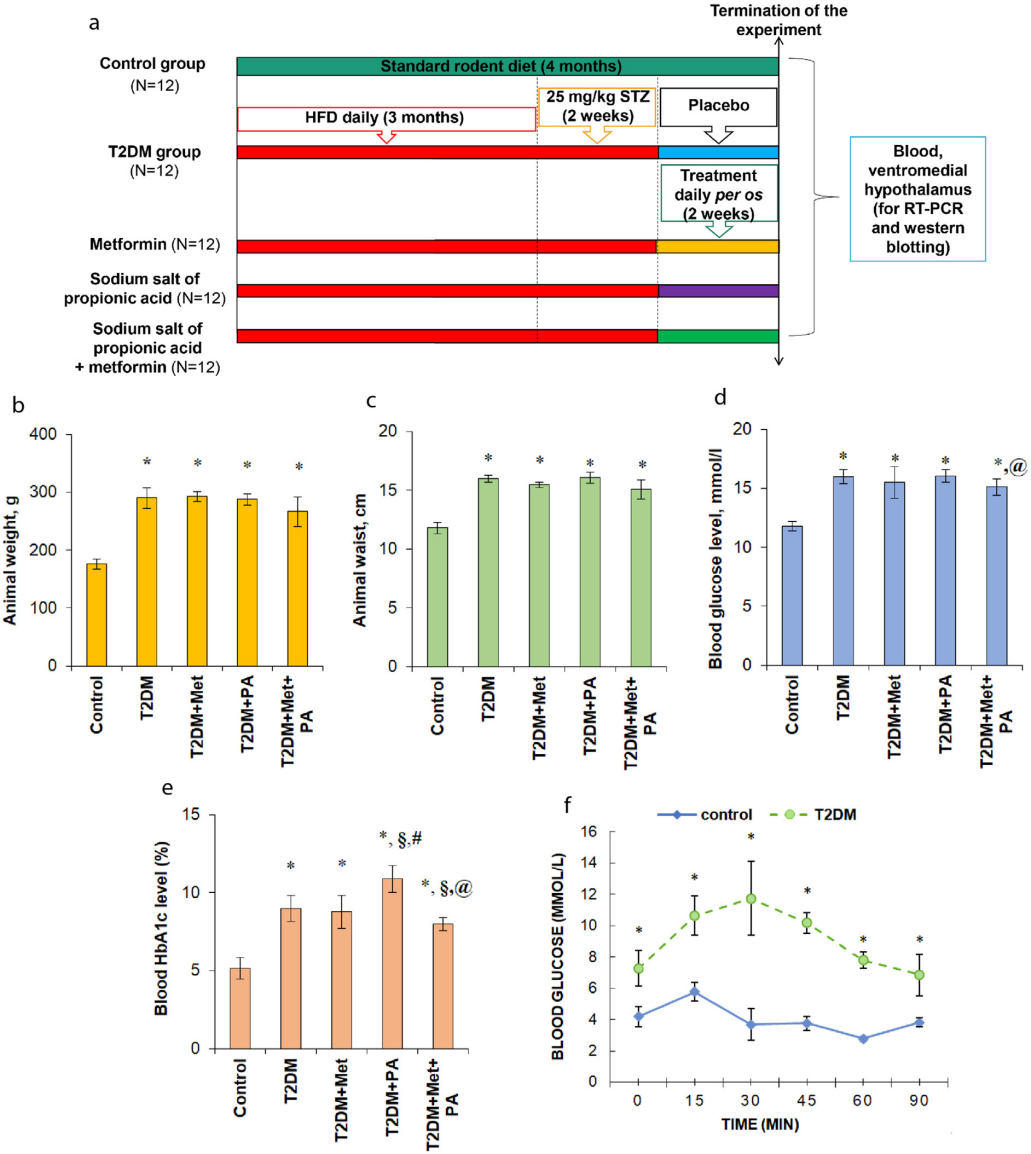
Experimental design and timescale (a). T2DM was developed after 3 months of high-fat diet with the following single injection of streptozotocin (25 mg/kg of b.w.). Each experimental group included 12 animals: 1) the untreated control group; 2) the group with experimentally induced T2DM; 3) the group that received anti-hyperglycemic agent metformin at a dose 60 mg/kg of b.w., for 14 days, orally on the background of T2DM; 4) the group that received sodium salt of propionic PA at a dose 60 mg/kg of b.w., for 14 days, orally on the background of T2DM; 5) the group that received metformin (60 mg/kg of b.w., for 14 days, orally) and PA (60 mg/kg of b.w., for 14 days, orally) on the background of T2DM. The body weight (b), waist (c), blood glucose (d) and glycosylated hemoglobin levels (e) of diabetic animals and after metformin and PA treatment. Intraperitoneal insulin tolerance test (f) on control rats (n = 6, blue solid line) and rats with T2DM (n = 24, green dashed line) before administration of substances. Blood samples were collected from the tail at the indicated time points and analyzed for glucose concentration (mmol/l). Values are given as mean ± SEM (n = 6); ∗p < 0.05 vs. control, ^§^p < 0.05 vs. T2DM, ^#^p < 0.05 vs. metformin administration, ^@^ p < 0.05 vs. PA administration.

The general experimental design is shown in Figure 1a. To induce T2DM, rats were fed a homogenous HFD mixture: standard rodent feed (34%), pre-melted fat from lard (45%), medical bile acids (1%, for natural emulsification of fat in the intestine of rats and improvement of its enteral absorption), and dry fructose (20%) [35]. Additionally, STZ administration was performed at a dose 25 mg/kg of b.w. [36, 37] once intraperitoneal after 3 months of HFD [38, 39], and after an injection, the standard rodent diet was returned for the next 2 weeks. After this period, all experimental animals with confirmed T2DM were randomly divided into 4 groups to treat them orally with placebo, metformin, PA and metformin + PA for two weeks [40]. In the T2DM placebo group rats were treated with the neutral saline solution at a similar volume as was the volume of other compounds. After two-week treatment on the background of T2DM, rats were sacrificed by a decapitation after administration of a lethal dose of sodium thiopental. Blood samples were collected for serum separation to determine the glucose and glycosylated hemoglobin (HbA1c) levels. For western blotting, RT-PCR and histological analysis, VMH samples were accurately dissected. The levels of autophagy markers LC3 and Beclin-1 on transcriptional and translational level were determined. The pro-apoptotic (Bax, caspase-3) and anti-apoptotic (Bcl-xl) markers were also measured (by RT-PCR, western-blot analysis and immunohistochemically) to characterize the process of apoptosis in the VMH of rats with T2DM, treated with metformin, PA and their combination. Moreover, the ultrathin sections of VMH after metformin and PA administration were examined with a transmission electron microscopy to visualize the possible morphological changes, including an assessment of the presence of apoptotic changes after metformin and PA treatment, as well as after their combined action.

### 2.3 Assessment of glycosylated hemoglobin level

The level of HbA1c was determined by the ion-exchange chromatography-spectrophotometry method using a standard kit «HEMOGLOBIN A1C-DIRECT» (HbA1C-DIR, #31047, Bio Systems, Spain). 50 μl of whole blood, that was collected previously in tubes with EDTA and 5 ml of distilled water, were lysed and then the mixture was applied on a microcolumn from the HbA1C-DIR kit. Filling buffer was used to perform the elution according to the manufacturer’s protocol. The optical density of eluates containing the HbA1c fraction was measured spectrophotometrically (on 415 nm). The optical density of the samples with total hemoglobin was also determined, and the results were expressed as a percentage (%) of the HbA1c fraction to the total hemoglobin content.

### 2.4 Slice preparation and electron microscopy

Rat VMH samples were fixed in 2.5% glutaraldehyde for 4 h and Millonig’s phosphate buffer (рН 7.4) followed with fixation by 1% osmium tetroxide for 1 h. After that, brief washing of slices in distilled water, dehydration in the graded series of ethanol solution (70%, 80%, 90%, 100%) and acetone were done. Then slices were poured into a mixture of epon-araldite (Epoxy embedding medium, #45345-250ML-F, Epoxy embedding medium, hardener DDSA, #45346-250ML-F, Araldite, #10951-250ML, Sigma, USA). Semi-thin and ultrathin sections were cut with the ultramicrotome “LKB III” (Sweden). To confirm that we work with the required brain area semi-thin sections (2–3 μm) were stained with methylene blue dye according to the Hayat method [41]; ultrathin sections (600–900 Å) were contrasted with 2% uranyl acetate solution and lead citrate. Transmission electron microscope “PEM-125” (Ukraine) with a magnification of 6000–20,000 times was used to examine the sections. By using ImageJ software we calculated the areas of the ER membranes and the ER cisterns in each cell.

### 2.5 RNA isolation and quantitative real-time (RT) PCR analysis

Total RNA from the ventromedial hypothalamus samples (30 mg) was isolated using GeneJET RNA Purification Kit (Thermo Fisher Scientific Inc., USA). mRNA concentrations and purity were determined by measuring the OD260/280 and OD260/230 ratios on DeNovix DS-11 FX+ (DeNovix Inc., USA). Purified RNA samples were reversely transcribed to cDNA using the RevertAid First Strand cDNA Synthesis Kit (Thermo Fisher Scientific Inc., USA). Quantification of mRNA levels was performed on 7500 Real-time PCR System (Life Technologies Corporation, USA). Target genes were amplified for 40 cycles using Maxima SYBR Green/ROX qPCR Master Mix (Thermo Fisher Scientific Inc., USA). A two-stage RT-PCR amplification reaction was performed under the following conditions: 95 °C for 10 min, followed by 40 cycles at 95 °C for 15 s, and at 60 °C for 50 s. The primer sequences are listed in Table 1 (designed using Primer BLAST software). Gene expression relative to the housekeeping gene *β-actin* was quantified as the fold change using the ΔΔCt method.

**Table 1:** The sequences of the primers.

Primer name	Forward primer sequence (5ˊ→3ˊ)	Reverse primer sequence (5ˊ→3ˊ)
*Bax*	*CACGTCTGCGGGGAGTC*	*CATCCTCTCTGCTCGATCCT*
*Bcl-xl*	*GGTCTCTTCAGGGGAAACTG*	*TCCAAAACACCTGCTCACTC*
*Beclin-1*	*GAGGAATGGAGGGGTCTAAGG*	*TGGCTGTGGTAAGTAATGGAGC*
*LC3*	*GTGCATTTGGCTTGGAAACTC*	*CCTTTTAGAGAAGGCAGCAGG*
*Gapdh*	*ACAACAACGAAACCTCCGTG*	*CACAAGCCCATTTCAGGGTA*
*β-actin*	*TGCAGAAGGAGATTACTGCCCTGG*	*GCTGATCCACATCTGCTGGAAGG*

### 2.6 Western blot analysis

Target protein levels (LC3, Beclin-1, Bcl-xl, Bax, caspase-3) were measured by western blotting. Protein extracts from frozen hypothalamus samples were prepared using a standard protocol with RIPA buffer as described previously [13]. Equal amounts of protein (30 μg per track) from each sample were loaded onto 10–15% PAAG (depending on the molecular weight of protein) for electrophoresis. Then protein samples were blotted on a nitrocellulose membrane (#HATF00010, 0.45 μm pore size, Merck Millipore, USA). Membranes were blocked with 5% non-fat milk in phosphate-buffered saline (PBS) with 0.05% Tween-20 (PBST) for 1h followed by an incubation overnight at +4 °C with primary antibodies against LC3 (1:1000, #sc-134226, Santa Cruz Biotechnology, USA), Beclin-1 (1:1500, #PA5-20171, Invitrogen, USA), Bcl-xl (1:1000, #PA5-21676, Invitrogen, USA), Bax (1:500, #МА5-14003, Invitrogen, USA), caspase-3 (1:2500, #ab208161, Abcam, UK) and tubulin (1:1000, T5168, Sigma-Aldrich, USA). Membranes were then washed and incubated with horseradish peroxidase (HRP)-conjugated secondary antibodies: anti-rabbit IgG (1:8000, #A0545, Sigma-Aldrich, USA) or anti-mouse IgG (1:5000, #ab97057, Abcam, UK). Thereafter, chemiluminescence development with p-coumaric acid (Sigma-Aldrich, USA) and luminol (Sigma-Aldrich, USA) was used to visualize protein bands. The relative levels of LC3, Beclin-1, Bcl-xL, Bax, and caspase-3 were normalized to tubulin content. Quantification of western blots was performed by measuring the optical densities of the respective bands using Gel-Pro Analyzer32 (v3.1) software.

### 2.7 Immunohistochemical staining and quantification

Immunohistochemical (IHC) studies were performed using polyclonal antibodies against Bax (#МА5-14003, Invitrogen, USA) and Bcl-xl (#PA5-21676, Invitrogen, USA) in dilution of 1:500. 3,3′-diaminobenzidine (DAB) staining and the EnVision FLEX detection system (Dako, Denmark) were used for visualization.

After rat sacrificing, a transcardial perfusion was performed 4% paraformaldehyde (PFA) in PBS (pH 7.4). The brain was postfixed in 4% PFA overnight, and after that, brain samples were dehydrated in the elevated concentrations of ethanol and embedded in paraplast (Leica-Paraplast Regular, 39601006, Leica Biosystems Inc., USA). VMH sections were obtained using a microtome Microm HM360 (Microm International GmbH, Germany) to cut slices of 5 μm thick and they were mounted on HistoBond®+ adhesive microscope slides (Marienfeld GmbH & Co. KG, Germany). All brain sections were within stereotaxic coordinates – 2.04...3.0 mm relative to Bregma point. To remove paraffin, slices were incubated in xylene and rehydrated in decreased concentration of ethanol solution (100%, 95%, 80% and 70%; for 2 min) and 2 steps of distilled water (for 2 min). Antigen unmasking was performed using 10 mM sodium citrate buffer (pH 6.0) (+98 °C, 30 min). After cooling to +65 °C, slides were washed for 1 min in 3 volumes of EnVision™ FLEX wash buffer. Endogenous peroxidase activity was blocked with the EnVision FLEX peroxidase-blocking reagent solution. Sections were incubated for 30 min in primary Bax or Bcl-xl antibodies (1:500), and then, with secondary HRP-conjugated antibody (EnVision FLEX/HRP) for 20 min. For visualization a mix of DAB (EnVision DAB + Chromogen) with EnVision FLEX substrate buffer was used for 10 min. Nuclei were stained with Gill III haematoxylin.

Brain sections were investigated using an Olympus microscope BX51 and documented by a digital camera Olympus C4040ZOOM with the software Olympus DP-Soft 3.2. For semi-quantitative evaluation, the intensity of staining was calculated according to the next scale: grade 0 for the absence or focal weak reaction; grade 1 for intense focal or diffuse weak reaction; grade 2 for moderate diffuse reaction; grade 3 for intense diffuse reaction; and grade 4 for the high intensity.

### 2.8 Statistical analysis

The data distribution was analyzed using the Shapiro-Wilk test. Statistical differences between the groups were analyzed by the one-way ANOVA test with the following Tukey post-hoc test. Results with p < 0*.*05 were considered statistically significant. The Pearson’s correlation coefficient (R) was calculated with a p-value 0.05 (95% confidence interval). All data were obtained based on two or three independent experiments and presented as mean ± SEM. Statistical analysis was performed using IBM SPSS Statistics, version 23.0 (SPSS Inc., USA).

## 3 Results

### 3.1 Effects of metformin and PA on animal body parameters, peripheral glucose homeostasis and insulin sensitivity

First, the verification of the development of T2DM after HFD was performed by measuring the animal body parameters (weight, g and waist, cm), the levels of glucose and glycosylated hemoglobin (HbA1c) in the serum of animals and also by applying an intraperitoneal insulin tolerance test. We observed that after T2DM induction, the body weight and the waist of diabetic rats was higher (1.65- and 1.35-fold respectively) compared with control animals (Figure 1b, 1c). Administration of metformin and PA, as well as their combination had no significant effects on body weight compared with T2DM group, however, the body weight after metformin, PA and their combined action was still higher compared with control animals by 67%, 63% and 51% respectively. The same effect of administration of compounds was observed for the waist parameter. Thus, animals in T2DM group showed a significant increase in the weight and waist compared with the control group reflecting the obesity development. In turn, PA and metformin treatment had no lowering effects on these parameters.

To further confirm the development of stable hyperglycemia, the levels of glucose and glycosylated hemoglobin in the blood were measured (Figure 1d, 1e). 4 weeks after the injection of STZ, fasting blood glucose was higher than in the control group by 1.36 times (Figure 1d) reflecting the development of hyperglycemia. After 2-week metformin and PA administration, the glucose level did not change compared with T2DM rats. Rats treated with metformin and PA group showed a slight reduction by about 14% of blood glucose level compared with rats treated only with PA.

Since HbA1c is an important marker of hyperglycemia, the next step was to estimate its level. We observed the correlation of HbA1c level with the glucose content in the blood. HbA1c level was higher by 74% in T2DM rats and by 70% in metformin group vs. control (Figure 1e). PA also had an increasing effect on HbA1c level compared with control animals (2.10-fold), with T2DM (by 21%) and metformin group (by 23.9%). In addition, we confirmed a slight reduction in HbA1c level in the group of rats with combined PA and metformin treatment by 13% vs. T2DM group and by 36.6% vs. PA group, however, it still remained higher than in control rats by 54%.

Thus, based on the observation of persistent hyperglycemia and an elevated HbA1c level as commonly used biomarkers for metabolic syndrome, we verified the development of experimentally induced T2DM in rats after 3-month HFD followed by a single STZ injection. Administration of PA and metformin had almost no effect on these parameters during the T2DM course.

Additionally, to assess the whole body insulin sensitivity, an intraperitoneal tolerance test (ipITT) was performed (Figure 1f). Animals were fasted for approximately 6 h (morning fast), fasted blood glucose levels (basal) were determined before an intraperitoneal insulin injection and after an intraperitoneal insulin injection glucose remaining in the circulation was also assessed within 15 min after the injection of insulin, blood glucose level in control animals increased by 1.3-fold (5.77 ± 0.29 mmol/l) compared with the basic control value (4.25 mmol/l) and went back to starting level after 30 min. At 60 min after the insulin injection, the glucose level was minimal (2.7 ± 0.08 mmol/l), and then after 15 min its level increased to 3.8 ± 0.14 mmol/l. In the T2DM group the basal glucose level (7.26 ± 0.45 mmol/l) was higher than in control rats. After 30 min it reached a maximum value – 11.73 ± 0.96 mmol/l, followed by a progressive decrease (to 10.17 ± 0.27 mmol/l on the 45th min and to 7.8 ± 0.21 mmol/l on the 60th min) reaching basal level after 90 min post injection (6.88 ± 0.55 mmol/l).

Thus, as expected in the course of T2DM model verification, rats from T2DM group demonstrated altered glucose tolerance and insulin sensitivity, which resulted in the impaired glucose homeostasis.

### 3.2 Ultrastructural changes of VMH in rats with T2DM after metformin and PA administration assessed by electronic microscopy

In the second part of this study, we addressed the following questions: (1) what were the ultrastructural changes in the VMH of diabetic rats and (2) what was the possible influence of metformin and PA administration on the general cell structure and organelles. For this purpose, an electron microscopic study was performed.

**Figure 2:**
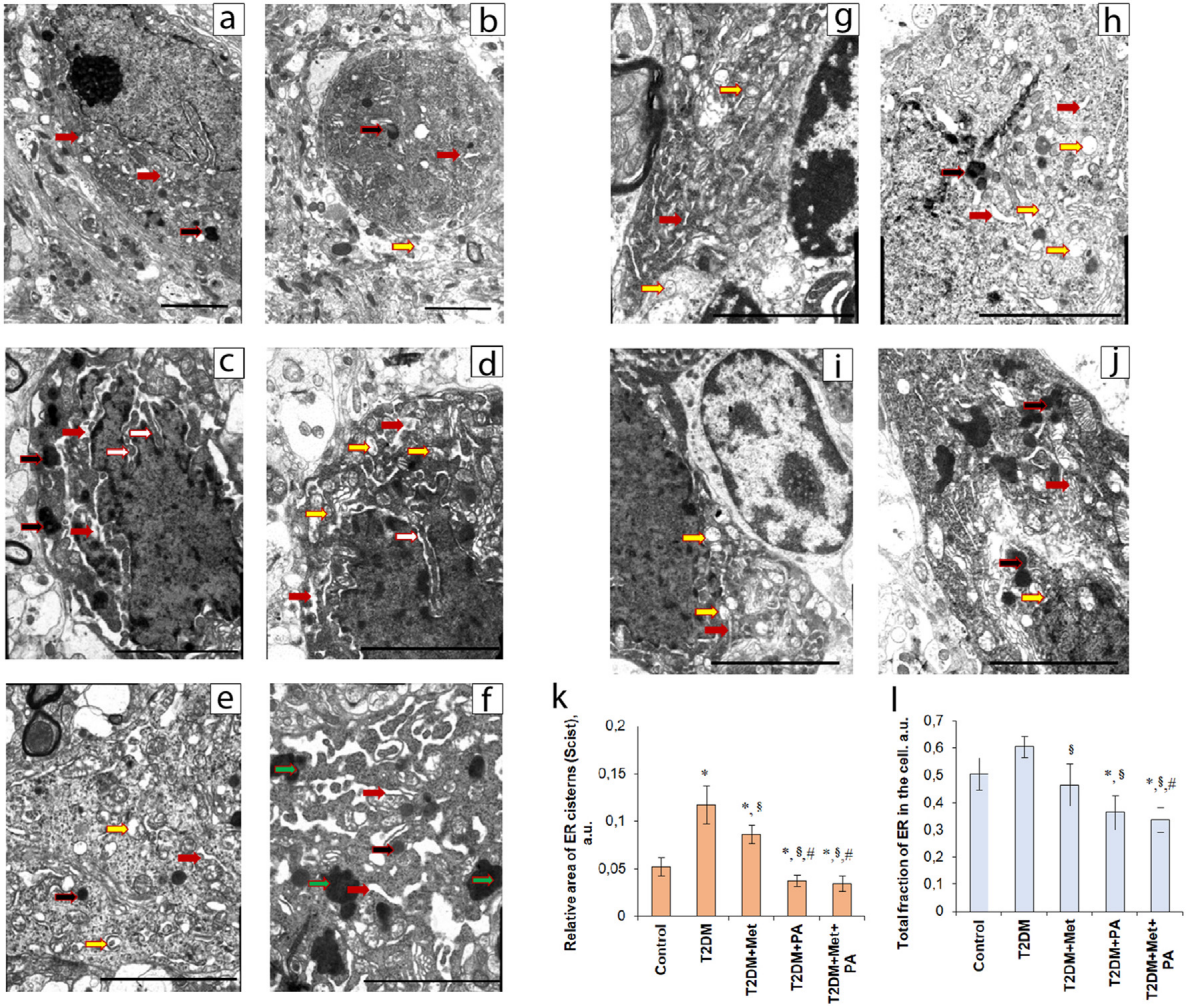
Ultrastructural changes of VMH neurons in T2DM rats and after treatment (electron microscopy). Gallery of micrographs obtained by a scanning electron microscope (SEM) (n = 12 neurons for each group): a, b – control rats (magnification– x4800); c, d – rats from T2DM group (magnification on C – x8000, D – x9600); e, f – T2DM rats treated with metformin (magnification – x9600); g, h – T2DM rats treated with propionic acid (magnification – x9600); i, j – T2DM rats treated with both metformin + propionic acid (magnification on a – x6400, b – x8000). The red arrows indicate the rough endoplasmic reticulum, yellow arrows – autophagosomes, black arrows – lysosomes, white arrows – deep invaginations of the nuclear membrane and green arrows – lipofuscin granules. Scale bar: 200 nm. Structural parameters of the ER after metformin and PA administration on the background of T2DM (k, I): ImageJ software was used to calculate the areas of the ER membranes and the ER cisterns (k) and its sum (total fraction of ER in cell, l) in each cell based on the electronic microscopic microphotographs. Values are given as mean ± SEM (n = 12); ∗p < 0.05 vs. control, ^§^p < 0.05 vs. T2DM, ^#^p < 0.05 vs. metformin administration, ^@^ p < 0.05 vs. PA administration.

According to electron microscopy examinations of VMH neurons of control rats (Figure 2a, 2b), we observed well-defined nuclei, with a small perinuclear space, which passed into ER. The Golgi complex and mitochondria were clearly defined and visible. In some neurons, single small autophagosomes and lysosomes were found.

We found that the electron density of the cytoplasm and nucleus was increased in most neurons (Figure 2c, 2d) in T2DM group compared with the control rats. In VMH neurons of diabetic rats, partial fragmentation of elements of the Golgi complex and an enlargement of the tubules of the granular ER were observed. Most mitochondria in VMH neurons showed signs of diabetes-associated structural damage: in particular, defragmentation, matrix edema and destruction of cristae. In a small percentage of neurons, considerable mitochondrial destruction was detected with the formation of mitophagosomes. Autophagosomes and lysosomes were also detected in the higher frequency compared with control (Figure 2c, black arrows – lysosomes; 2D, yellow arrows – autophagosomes). Most of the nuclei of VMH neurons in T2DM group underwent pyknotic changes with the appearance of deep invaginations of the nuclear membrane (Figure 2c, 2d, white arrows) and, in addition, fragmentation of nuclei was observed in single cells. Neurons with apoptotic changes, axon and dendritic loss, and alignment of the plasma membrane in VMH of T2DM rats were predominated. “Light” neurons were practically not detected. Swelling of synaptic terminals with the loss of vesicles was detected in the neuropil of VMH in the T2DM group. Astrocytes surrounding of VMH neurons showed signs of cytoplasmic edema, and a decrease in the total number of organelles was observed.

Metformin administration slightly amended diabetic-induced cellular changes in VMH. After metformin treatment, neuronal Golgi complex was clearly visible, the ER cisterns in most cells did not differ from those in untreated control rats (Figure 2e, 2f). A clear outline of the tubules of the granular ER with the abundance of ribosomes on their surface was also noticed, however, the structure of mitochondria was more preserved than in the T2DM group. Concerning the ER cisterns, their volume has been reduced after metformin compared with the T2DM group. In addition, administration of metformin on the background of T2DM was accompanied by an accumulation of lipofuscin granules in the cytoplasm of VMH neurons (Figure 2f, green arrows), as well as the presence of reactive astrogliosis, characterized by an enhanced proliferation and hypertrophy of the surrounding astrocytes. Moreover, an accumulation of autophagosomes, an increase in the number of lysosomes, axoplasm swelling, and damage of the myelin sheath as it was observed in the T2DM group, were still detected in VMH neurons of metformin-treated rats.

Treatment of T2DM rats with propionic acid was accompanied by an improved visualization of the outline of intracellular membrane structures in VMH neurons, mostly similar to control group (Figure 2g, 2h). Tubules of rough ER showed the high level of preservation, clearly visible and well defined ribosomes were located on ER surface without accumulating in the cytoplasm. We observed that the total volume of ER cisterns was relative to the control group (Figure 2g, 2h). The interesting point that observed in T2DM + PA group was the identification of two types of autophagosomes in VMH neurons: primary with a double-layered membrane and secondary with a single-layered membrane containing cellular detritus, that might be considered a sign of the accumulation of aggregates of misfolded proteins. Administration of PA to animals with T2DM visually reduced the number of pre-apoptotic and apoptotic “dark” neurons with pyknotic nuclei compared with T2DM. In the T2DM + PA group, “light” neurons with the large light nuclei were predominated and an increased number of deep nuclear invaginations was detected as compared with the control group. In a small number of neurons, a partial enlargement of the perinuclear space was observed. Altogether, these observations may indicate the development of the compensatory and adaptive processes aimed at increasing the area of contact between the nucleus and the cytoplasm. After PA administration, astrocytes were with no signs of swelling. Thus, according to these observations, PA probably in more extent than metformin prevented diabetes-induced alterations of VMH cytoarchitectonics.

In the group with the administration of metformin and propionic acid (Figure 2i, 2j), we detected that their concurrent administration led to the elevated quantity of microglial cells in the VMH region of the hypothalamus. Additionally, combined administration of metformin and PA was accompanied by a slight decrease in the total amount of pre-apoptotic and apoptotic neurons, in comparison with the T2DM + metformin and T2DM + PA groups. In “light” neurons, ER tubules were well-visualized, however, with a small number of ribosomes (Figure 2i, 2j, red arrows). In “dark” neurons, an enlargement of ER cisterns and vesicles was observed. In VMH neurons of T2DM rats treated with the combination of drugs, the number of swollen mitochondria, lysosomes (Figure 2j, black arrows), and lipofuscin granules was increased compared with the control animals, however, their quantity was less than in the T2DM group and the metformin group. The nuclei of “light” neurons demonstrated significantly more invaginations of the nuclear membrane compared with the “dark” neurons. The interesting point observed here was that astrocytes were less swollen near “dark” neurons as compared with the T2DM group.

After considering and comparing all cytoarchitectonic differences on VMH cells based on the results of electron microscopy, we can state that diabetes-related changes in VMH neurons were manifested in the form of fragmentation of membrane organelles including mitochondria, an increase in the number of cells with apoptotic changes and neuropil edema. These changes were improved to a different extent after the administration of drugs that, in general, partially counteract ER damage, prevented mitochondrial swelling, reduced the number of apoptotic neurons and enhanced the glial response.

Additionally, since an increasing evidence has shown a strong link between ER stress and the pathology of obesity and T2DM that may lead to the leptin and insulin resistance, we performed a quantitative assessment of the ER morphometric parameters in the hypothalamus of rats with T2DM and after metformin and propionic acid administration (Figure 2k, 2I). An overall fraction of ER in the cell was quantified as the sum of the relative areas of ER membranes and cisterns (Figure 2I). Figure 2I shows a 2.25-fold enlargement of the ER cistern areas in the T2DM group as compared with the control ER parameters and the slight tendency to an increased total ER fraction (by 19%), however, without statistically significant difference (Figure 2k, 2I). Administration of compounds studied caused the similar decreasing effect on the parameters of relative area of ER cisterns and total ER fraction. Metformin reduced the relative area of the ER cisterns in T2DM rats by 36% (vs. T2DM), that was confirmed by observations on the electron microscopy images presented previously, however, this parameter was still by 65% higher than in the control group. Total fraction of ER in the cell demonstrated no significant changes in the metformin group compared with the control group, however, it was lower than in T2DM rats (Figure 2k). РA administration had a reducing effect on the area of ER cisterns (by 40.5% vs. control, by 316% vs. T2DM, by 232% vs. metformin) and on the total fraction of ER in the cell (by 39% vs. control and by 66% vs. T2DM) in VMH neurons. Finally, PA and metformin altogether caused the substantial decline in the relative area of ER cisterns (by 53% vs. control, by 344% vs. T2DM, by 253% vs. metformin) and the total fraction of ER in the cell (by 50% vs. control, by 80% vs. T2DM, by 38% vs. metformin) (Figure 2I). Thus, under the influence of compounds on the background of T2DM, an interesting point should be emphasized: after the T2DM-associated elevation, the following ER characteristics as relative area of ER cisterns, and the total fraction of ER in the cell have been reduced to a different extent not only when comparing with the T2DM group but also vs. control values after the treatment with metformin, PA and their combined exposure.

### 3.3 The influence of metformin and PA on the apoptosis/autophagy markers in the VMH of rats with T2DM

**Figure 3:**
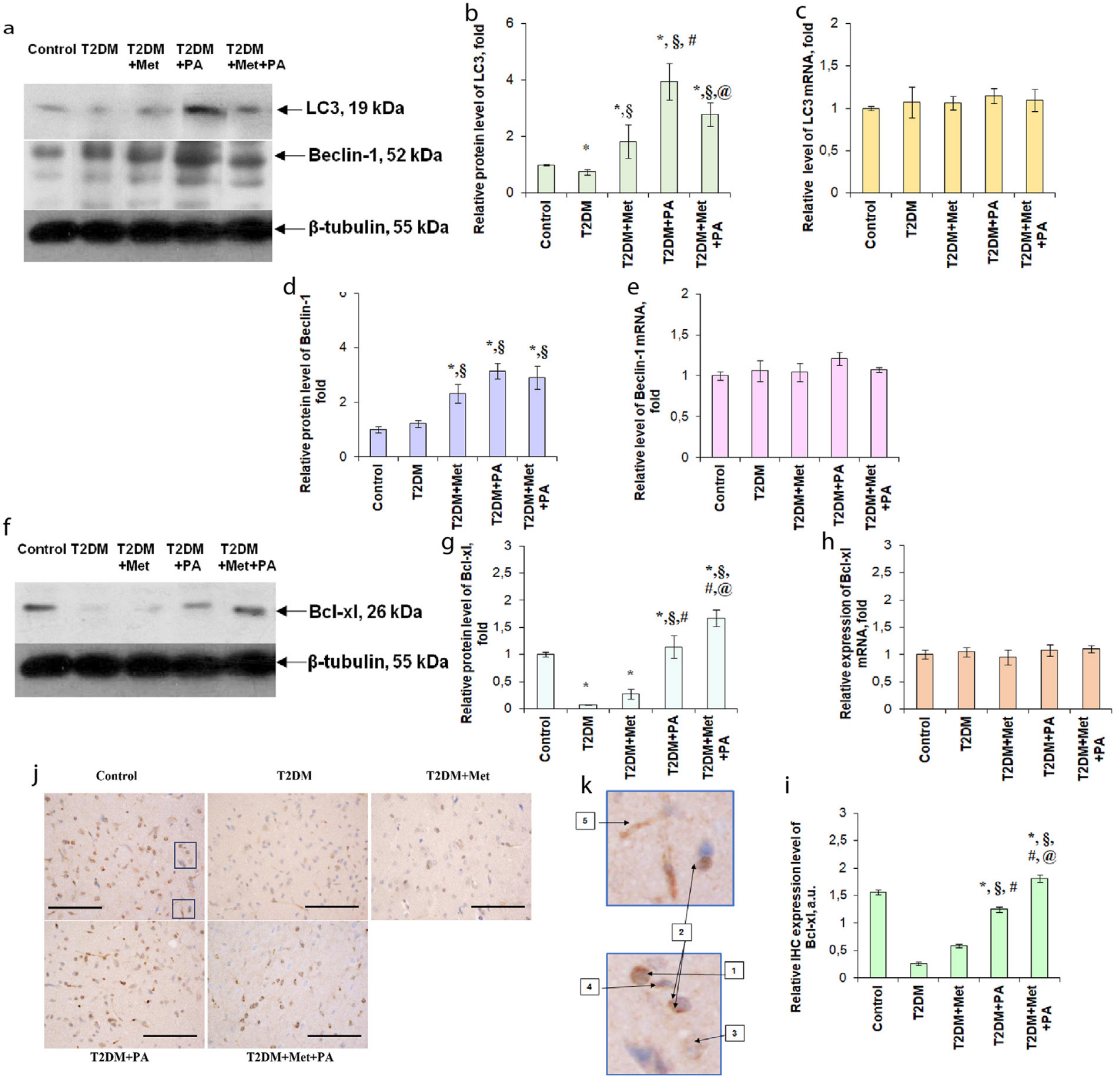
Effects of metformin and PA treatment on LС3, Beclin-1 and Bcl-xl mRNA expression and protein content in VMH of T2DM rats. Immunoblotting analysis: representative immunoblots are shown (a, f) and level of studied proteins was quantified using tubulin as a loading control for VMH lysates. The uncropped versions of the LС3, Beclin-1 and Bcl-xl immunoblots are included in the Supplementary_Material_1. The bar graphs of LС3 (b), Beclin-1 (d) and Bcl-xl (g) are presented as means ± SEM (n = 6/group). Quantitative real-time PCR analysis of LC3 (c), Beclin-1 (e) and Bcl-xl (h) mRNAs expression in rat VMH: data were normalized to both β-actin/GAPDH and pooled from three independent experiments (n = 6 rats/group). Immunocytochemical analysis of Bcl-xl-positive VMH cells: representative histogram (i) and images (j) are shown. DAB (3,3′-diaminobenzidine) staining was use to visualize Bcl-xl-positive cells, Hematoxylin Gill was used for nuclear staining. Scale bars indicate 150 μm (magnification х400). Immunocytochemical analysis of Bcl-xl-positive VMH cells of the control rats (k): 1 – moderately and/or intensively labeled macules; 2 – macules near the nucleus; 3 – neurons with diffuse Bcl-xl expression in the cytoplasm; 4 – glial cells with Bcl-xl expression; 5 – Bcl-xl staining in the endothelium of the blood capillaries of the VMH (magnification of fragment х3200). All data are shown as means ± SEM; ∗p < 0.05 vs. control, ^§^p < 0.05 vs. T2DM, ^#^p < 0.05 vs. metformin administration, ^@^ p < 0.05 vs. PA administration.

Since we detected an elevated number of autophagosomes in T2DM rats after treatment, we aimed to assess the autophagy process based on the measurement of two markers – LC3 and Beclin-1. Thus, LC3 is considered as one of the autophagosomal markers in mammals, and has been frequently used to study an autophagy under the neurodegenerative and neuromuscular diseases, tumorigenesis, bacterial, and viral infections [42]. In the T2DM group LС3 protein level was reduced by 25% compared with the control group (Figure 3a, 3b, Supplementary_Material_1), suggesting a slight inhibition of an autophagy process. Metformin, PA and their concurrent administration to T2DM rats significantly increased LС3 protein content by 1.82, 3.95 and 2.79 folds respectively. However, no meaningful between-group differences in the LC3 mRNA level were found, suggesting that all changes occurred on translational level (Figure 3c).

Another important marker that play a central role in the regulation of an autophagy and membrane trafficking in physiological and pathological processes, Beclin-1, was measured to further confirm the activation of an autophagy after the compound administration. Moreover, since Beclin-1 is localized primarily within cytoplasmic structures, including the ER, mitochondria and the perinuclear membrane [43], and we observed T2DM-induced changes in the architectonics of these cell structures, it would be interesting to juxtapose it with the fluctuations of Beclin-1 protein level. Interestingly, no meaningful differences in Beclin-1 content were observed between the control and the T2DM group. However, after metformin (by 2.33 folds) and PA (by 3.15 folds) administration and their combination (by 2.91 times) Beclin-1 level was increased, that was in line with the LC3 protein level fluctuations (Figure 3a, 3d, Supplementary_Material_1). Beclin-1 mRNA levels remained unchanged in all 5 groups of animals (Figure 3e), confirming the hypothesis, that all T2DM-induced changes occurred on the translational level. Thus, the similar increasing effects of PA and metformin on the autophagy markers were detected, suggesting the possibility of these compounds to activate the autophagy process in VMH to a different extent.

Since Beclin-1 can also be involved in the autophagic suppression of apoptosis [44] and considering the strong link between Beclin-1 and Bcl2/Bcx-xl complex, the next step was to estimate the state of Bax/Bcl-xl apoptotic regulatory axis. It is known that Bcl-xl exerts apoptosis-inhibiting effects, therefore we examined its content by different methods. We found that experimentally induced T2DM was accompanied by a strong 10-fold decrease in the expression of the apoptosis-inhibiting protein Bcl-xl in the VMH (Figure 3f, 3g, Supplementary_Material_1). Metformin administration led to a slight elevation of Bcl-xl protein content compared with the T2DM group, however, PA alone and PA together with metformin exerted more pronounced increasing effects on the expression of Bcl-xl protein, which was even higher than in the VMH of the control animals (by 14% and 67% vs. respectively). Bcl-xl gene expression almost did not change in the groups compared with the control level (Figure 3h).

**Figure 4:**
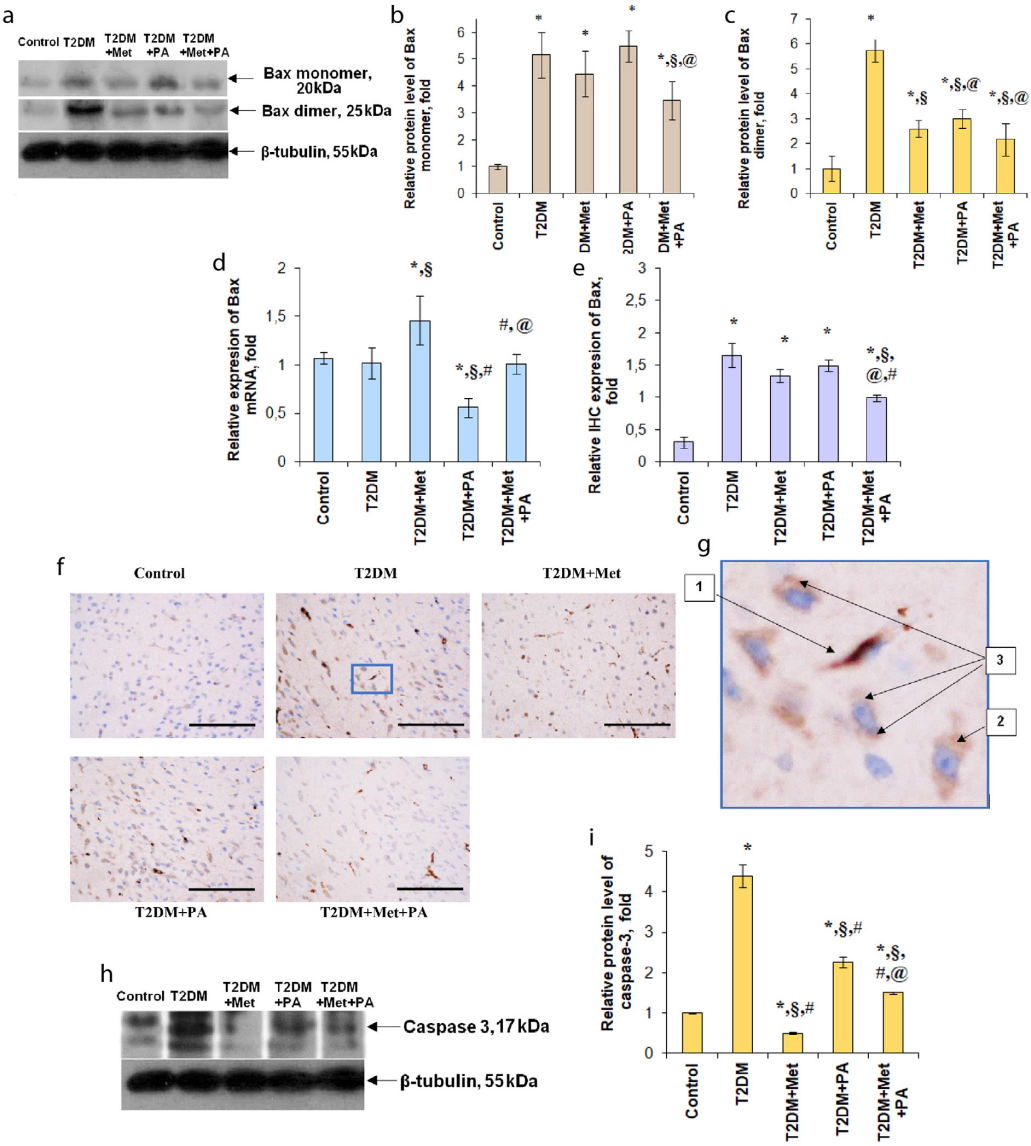
Effects of administration of metformin and PA on the levels of apoptotic and anti-apoptotic markers. Immunoblotting analysis of Bax in rat VMH: representative immunoblots show (a) the monomeric and dimeric forms (b, c), that were quantified using tubulin as a loading control for VMH lysates. The bar graphs of Bax protein content (b, c) are presented as means ± SEM (n = 6/group). RT-PCR of Bax in rat VMH (d): data were normalized to both β-actin/GAPDH and pooled from three independent experiments (n = 6 rats/group). Immunocytochemical analysis of Bax-positive VMH cells: representative histogram (e) and images (f) are shown. DAB (3,3′-diaminobenzidine) staining was used to visualize Bax-positive cells, Hematoxylin Gill was applied for nuclear staining. Scale bars indicate 150 μm (magnification х400). Bax-positive VMH cells of the T2DM rats (g): 1 – blood capillaries of the VMH with the high Bax expression; 2 – VMH neurons with cytoplasmic expression of Bax; 3 – glial cells demonstrated small diffusely labeled areas of the cytoplasm located near the nucleus (magnification of fragment х3200). Caspase-3 protein level after metformin and PA administration on the background of T2DM: immunoblotting analysis of caspase-3 in rat VMH; representative immunoblots are shown (h) and quantified using tubulin as a loading control for VMH lysates. The uncropped versions of the Bax and caspase-3 immunoblots are included in the Supplementary_Material_2. The bar graph of caspase-3 (i) is presented as mean ± SEM (n = 6/group); ∗p < 0.05 vs. control, ^§^p < 0.05 vs. T2DM, ^#^p < 0.05 vs. metformin administration, ^@^ p < 0.05 vs. PA administration.

Immunohistochemical staining of the VMH sections with Bcl-xl antibody and the following semi-quantitative assessment of the obtained IHC images confirmed changes observed on the protein level (Figure 3i). VMH region of the control rats has demonstrated a concise Bcl-xl expression in the predominant number of neurons and glial cells (Figure 3k). In VMH neurons, the Bcl-xl protein was visualized as moderately and/or intensively labeled macules (Figure 3k-1) near the perinuclear space (Figure 3k-2). A small number of VMH neurons of the control group with a low level of Bcl-xl expression showed its diffuse localization in the cytoplasm (Figure 3k-3). In glial cells, Bcl-xl was observed as granules of different sizes in the perinuclear space (Figure 3k-4). Interestingly, it should be noted that a moderate Bcl-xl staining was observed in the endothelium of the blood capillaries of the VMH region (Figure 3k-5).

In the T2DM group, there was a significant decrease in Bcl-xl expression in neurons, glial cells, and an endothelium of VMH (Figure 3i). In most cells, Bcl-xl expression was not detected, or a weak diffuse staining was determined in the cytoplasm of VMH neurons.

Metformin administration led to a mild increase in Bcl-xl expression in VMH neurons and, to a lesser extent, in glial cells and vascular endothelium (Figure 3j, 3i). Bcl-xl protein in VMH neurons appeared as the stained macules in the perinuclear space, however, in separate VMH neurons, a weak staining of the entire cytoplasm was observed. In turn, PA administration induced a further increase in Bcl-xl expression in the VMH, almost to the control values (Figure 3j, 3i). There was a significant elevation of the number of neurons with well-stained macules, which were larger than in the control group. Glial cells also had enlarged immunoreactive granules with more intense staining than in the control group. The expression of Bcl-xl in the vascular endothelium also increased in comparison with the VMH of rats of the T2DM group, but was still lower than in control samples. Finally, co-administration of metformin and PA induced the more pronounced elevation of Bcl-xl expression in VMH, even when comparing with control samples (Figure 3j, 3i). Most neurons had intensively stained macules, the size of which was larger than in the control rats. A significant Bcl-xl increase was also detected in glial cells compared with all groups; however, Bcl-xl expression in the vascular endothelium was similar to the control animals. Thus, after inducing T2DM, an activation of anti-apoptotic protein Bcl-xl was decreased, probably contributing to the enhancement of apoptosis in VMH neurons. Treatment with PA and metformin led to an increase of Bcl-xl protein level in all types of cells, and the combination of metformin and PA exerted the most effective anti-apoptotic influence.

Bax is a well-known regulatory protein due to its apoptosis-promoting biologic functions. Moreover, since Bax is a key regulator of apoptosis that mediates the release of cytochrome c to the cytosol via an oligomerization from a monomer to a dimer in the outer mitochondrial membrane before pore formation [45], it is important to determine its monomer and dimer forms. Western blot analysis revealed that the content of Bax-monomer in the T2DM group was higher by 5.16 times than in the control animals (Figure 4a, 4b, Supplementary_Material_2).

The same pattern was observed after the separate metformin and PA administration – Bax-monomer level was as in the VMH of T2DM animals (Figure 4a, 4b, Supplementary_Material_2). Only the concurrent combination of metformin + PA decreased the T2DM-induced monomeric Bax level by 1.5-fold vs. T2DM. At the same time, when compared with the T2DM-associated elevation, Bax dimeric form was reduced after all types of treatments (metformin – by 2.2-fold, PA – by 1.9-fold, combined treatment – by 2.7-fold) (Figure 4a, 4c, Supplementary_Material_2). Interestingly, in the VMH of the T2DM rats, Bax mRNA expression remained unchanged compared with the control rats, however, the administration of metformin contributed to a significant increase in its expression compared with the T2DM group, and even compared vs. control (by 1.45 times, Figure 4d). These data are not unexpected, since it is known that metformin may significantly elevate p53 and Bax levels and induce apoptosis in human MCF-7 breast cancer cells via targeting the extracellular signal-regulated kinase 1 (ERK) signaling [45]. In contrast, PA administration reduced Bax mRNA level compared with the T2DM rats by 1.81 times (Figure 4d), therefore, when we studied the combined treatment, we observed the compensation of an increasing effect of metformin on Bax content and a decreasing action of PA, resulted in the Bax levels that was relative those in the control and the T2DM groups.

In addition, the results of the IHC semi-quantitative analysis of Bax expression in VMH confirmed the changes in Bax protein content after the metformin and PA administration on the background of T2DM. VMH region of control animals showed different levels of Bax expression depending on the cell types. Endothelial Bax expression was at low and moderate levels, in neurons and glial cells – in small amounts both in the cytoplasm and in the nuclei (Figure 4f). T2DM caused a significant increase in Bax expression in VMH (Figure 4e, 4f, 4g), mostly in the endothelial cells (Figure 4g-1). In VMH neurons, there was an elevation of Bax expression in the cytoplasm (Figure 4g-2), however, not in the nucleus. Small diffusely stained areas in the perinuclear space were found in glial cells (Figure 4g-3). Metformin treatment slightly decreased Bax expression in comparison with the T2DM group (Figure 4e). Diffuse Bax staining in the cytoplasm of VMH neurons was observed. Bax-positive glial cells showed the moderate nuclear localization of Bax, however, it was rarely observed in the cytoplasm and in the Golgi complex. Propionic acid did not affect the level of Bax expression in VMH compared with T2DM group (Figure 4e, 4f). In the endothelium of blood vessels, expression of Bax protein was from moderate to high level. A significant part of VMH neurons after PA action showed the moderate Bax expression in the cytoplasm. In glial cells, Bax was localized in the perinuclear space in the form of granules. Unexpectedly, co-administration of metformin and PA led to a significant Bax decrease in VMH (Figure 4e, 4f); however, it was still 3-fold higher than in the control animals. Interestingly, in endothelial cells, Bax expression was slightly decreased compared with the T2DM group. In turn, the number of intensively stained neurons was significantly higher than in the control rats, but an intensity of labeling was lower than in the metformin and PA groups. In addition, after combined treatment with metformin and PA, Bax was localized in the perinuclear space of the glial cells.

Thus, the development of T2DM was associated with an elevated Bax expression in VMH cells, especially in the endothelium of blood vessels, that may indicate an activation of the Bax oligomerization process. In turn, this triggers a pro-apoptotic effect that may contribute to the development of the diabetes-associated brain endothelial dysfunction. Combined treatment with metformin and PA was effective in the inhibition of pro-apoptotic factor Bax and in the activation of anti-apoptotic factor Bcl-xl that may mediate their neuroprotective effect on the background of T2DM.

The next part of the study was to estimate the occurrence of apoptosis based on the level of an another trigger apoptotic marker – caspase-3, an executioner caspase whose activation by proteolytic cleavage often plays a role in apoptosis [47]. Neuron injury, caused by an excessive release of the excitatory neurotransmitter glutamate, leads to the widespread activation of caspase-3 [48], particularly in neurons undergoing apoptosis, but primarily in the nuclei of astrocytes, that are not undergoing apoptosis. We found that the caspase-3 protein level in VMH of the T2DM rats was dramatically increased by 4.4-fold compared with the control rats (Figure 4h, 4i, Supplementary_Material_2). The administration of the studied substances may have the possible anti-apoptotic and neuroprotective effects, that are expressed in diminishing caspase-3 level after metformin (by 8.8 times), after PA treatment (by 1.94 times), and expectedly when their action was combined – by 2.93 folds vs. T2DM. However, caspase-3 level after all treatments was still 1.5-fold higher than in the control group.

To summarize, in animals with experimentally induced T2DM a critical increase in the level of caspase-3 was observed, that might reflect the high intensity of apoptotic events in VMH. The combination of metformin and PA was effective against apoptosis, however, the caspase-3 content remained by 6 times higher compared with the control group.

Last, to further characterize apoptosis/autophagy process and find the representative correlation links between all studied parameters, we calculated the Pearson’s ratio (Table 2). Reasonably, we found a high positive correlation between the autophagy markers Beclin-1 and LC3, Beclin-1 and anti-apoptotic factor Bcl-xl, and between Bcl-xl and LC3 as well. The level of pro-apoptotic factor Bax was linked with caspase-3 content by the high positive correlation, however, caspase-3 content was negatively correlated with Bcl-xl, that was in agreement with their main functions in the cell.

**Table 2:** Correlation (Pearson’s ratio) matrix highlighting the link between apoptosis/autophagy markers in rat VMH.

	Beclin-1	Caspase-3	Bcl-xl	Bax dimer	LC3
Beclin-1	1	-,042	,537∗∗	,061	,781∗∗
Caspase-3	-,042	1	-,417∗	,779∗∗	-,109
Bcl-xl	,537∗∗	-,417∗	1	-,344	,477∗
Bax dimer	,061	,779∗∗	-,344	1	-,214
LC3	,781∗∗	-,109	,477∗	-,214	1

Note: ∗ - р< 0.05; ∗∗ - р <0.01.

## 4 Discussion

Since the balance between the survival and loss of hypothalamic neurons may have an impact on the coordinated control of feeding [49], the present study aimed to evaluate whether the HFD can induce apoptosis and/or autophagy of cells in the VMH area of hypothalamus, and to investigate how possible neuroprotective options in combination with metformin may influence cell death processes.

Autophagy is a catabolic pathway activated by cellular stress, that results in cellular adaptation, survival or cell death [50]. Despite the mechanisms of autophagy and apoptosis have differences, some proteins impact both processes and these switch points may be considered the potential targets for therapeutic interventions. Thus, the interconnected molecular regulators between autophagy and apoptosis serve as switching points critical to the cell fate [51]. For instance, anti-apoptotic Bcl-2 and Bcl-xl inhibit both autophagy and apoptosis through interacting respectively with Beclin-1 and Bax/Bak using their Bcl-2-homology (BH)-3-binding pockets [52, 53]. Previous studies showed that Bcl-2/Bcl-xl phosphorylation may be a regulatory switch between autophagy and apoptosis [54]. Since defects in the process of an autophagy may underlie the development of neurodegenerative diseases [55], our study was performed to investigate the crosstalk between autophagy and apoptosis in VMH after the treatment of metformin and PA by studying apoptosis-promoting (Bax, caspase-3), apoptosis-inhibiting (Bcl-xl) and autophagy-associated (LC3, Beclin-1) proteins in VMH of rats with T2DM and after the treatment.

The main question that arises from our study is what are the possible options to correct diabetes-induced pathological changes in VMH. Therefore, in the present study we investigated the action of metformin and sodium salt of propionic on autophagy and apoptosis in hypothalamus as possible neuroprotective strategies. As a comparison drug and a cornerstone of oral antidiabetic treatment, we chose metformin; in order to test another approach for the treatment of the T2DM-associated neuropathy influencing gut-brain axis, we considered the enteric-soluble SCFAs such as PA due to its normalizing effect on fasting blood glucose, body weight and insulin tolerance [56]. However, the direct effect of the PA usage as an additional therapy on the neurons of the VMH in T2DM should be elucidated, therefore, we focused on the complex approach in order to visualize cellular changes in VMH in combination with the molecular impairments in the regulation of apoptosis/autophagy interplay.

HFD-fed rodents can serve as a model to predict and/or test, the metabolic activity of metformin and PA. As expected with the development of HFD-induced T2DM, we observed the substantial changes in VMH: enlarged tubules and cisterns of rough ER in most neurons, an accumulation of fragmented mitochondria with signs of cristae destruction and matrix swelling, pyknotic nuclei. Altogether, these changes resulted in an increased number of apoptotic neurons and dendritic loss in T2DM group. These observations on the cellular level were accompanied by an imbalance between pro- and anti-apoptotic molecular regulators: first, by a dramatic elevation of pro-apoptotic factors Bax, that may translocate to the outer mitochondrial membrane and undergo oligomerization, leading the release of apoptogenic factors such as cytochrome c, and caspase-3, that confirmed cellular changes observed by an electron microscopy; second, by a slight decrease in autophagy marker LC3 and anti-apoptotic factor Bcl-xl, that may contribute to the inhibition of autophagy. Thus, the development of T2DM was associated with an activation of apoptosis and an inhibition of autophagy that may underlie pathological processes in hypothalamus and, as a result, an impaired glucose homeostasis and the development of neuropathy. Our results were in line with the study showing that the fat-rich diet induced neuronal apoptosis [57]. Moreover, an induction of cell death in the hypothalamus and/or the whole brain can mimic metabolic inflammation that may mediate the development of central leptin and insulin resistance, resulting in a broad range of metabolic disorders including overeating, glucose intolerance and hypertension.

Our data partially confirmed the pre-study hypothesis, that the administration of drugs may reduce to varying degrees the damaged manifestations in the hypothalamus by lowering mitochondrial swelling, diminishing the number of apoptotic neurons on the background of the enhanced glial response. This was in line with the available data that metformin exerted neuroprotective action in primary cortical neurons [58]. However, to our knowledge, this is the first study related to the possible influence of metformin on apoptosis/autophagy switch in VMH on the background of T2DM. Metformin administration resulted in swelling of myelinated fibers*,* an accumulation of autophagosomes and lysosomes, as well as reduced volume and relative area of the ER cisterns compared to those in the T2DM animals. Elevated number of lysosomes and autophagosomes as signs of autophagy were associated with an elevation of the levels of anti-apoptotic protein Bcl-xl, autophagy markers LC3 and Beclin-1, and a decrease in pro-apoptotic proteins Bax and caspase-3 compared with the T2DM animals. However, the administration of metformin was accompanied by undesirable effects manifested as the clearly visible accumulation of lipofuscin granules in the cytoplasm of VMH neurons and the development of reactive astrogliosis. Lipofuscin granules are a hallmark of aging and their progressive accumulation impairs adequate stress response, results in inflammasome activation and secretion of inflammatory cytokines, and increases an oxidation of unsaturated fatty acids that altogether may mediate the development of neuronal degeneration. Furthermore, an experimental work on *Caenorhabditis elegans* and human primary cells demonstrated that the treatment with metformin caused age-related disruptions in conservative metabolic pathways and led to the development of mitochondrial dysfunction [59]. Thus, despite the effective correction of hyperglycemia, metformin monotherapy did not prevent the development of diabetes-induced hypothalamic impairments. Therefore, our next step was to test PA as an additional neuroprotective therapy to correct neurodegenerative diabetes-induced changes and mitigate the potential negative effects of a single metformin treatment on the state of VMH.

PA action on VMH architectonics was similar to effects that were observed in the metformin group, but in a greater extent. We observed a significant number of autophagosomes and a decrease in the number of apoptotic cells compared with the T2DM group. It was in line with a substantial elevation on LC3 and Beclin-1 levels, suggesting an activation of autophagy process, that was correlated with an increase in Bcl-xl content up to control values. However, despite that fact that PA administration led to a decrease in the number of apoptotic “dark” neurons with pyknotic nuclei compared with the T2DM group, unexpectedly, pro-apoptotic protein Bax (both dimer and monomer forms) as well as caspase-3 levels were upregulated after PA administration, showing the tendency to the launch of apoptosis, however, it was not reflected at the cellular level with apoptotic signs. Bax may interact with Beclin-1 and inhibit an autophagy [50], thus, may represent a new crosstalk point. Moreover, it is known that Bax may oligomerize and trigger mitochondria-dependent apoptosis, however, we observed an elevation of the only Bax monomer, while Bax dimer was downregulated compared with the T2DM group, that may explain the absence of the signs of apoptosis and mitoptosis during the electron microscopy examinations. In addition, the positive point needed to be emphasized here is that the difference compared with the metformin group was that astrocytic glia demonstrated no signs of swelling. Generally, our previous hypothesis that PA may exert neuroprotective effect by activating anti-apoptotic mechanisms in neurons and stimulating an autophagy [13] as an important protective way to prevent an accumulation of misfolded proteins was decisively confirmed in this study.

Finally, the interesting data were obtained on the diabetic rats after combined administration of metformin and propionic acid. From the current results, as well as from our previous study [13], we expected that the combination of metformin and PA would be more effective than their separate usage. Unexpectedly, in the group with the combined treatment we observed an increased amount of microglia and a significant reduction of the percentage of “light” neurons compared with the control group. Additionally, administration of metformin and PA was accompanied by a slight decrease in the number of pre-apoptotic and apoptotic neurons, in comparison with the T2DM + metformin and T2DM + PA groups. These observations correlated with a decrease in caspase-3 and Bax monomer and dimer levels, confirming diminished apoptotic changes compared with other treatment groups. Considering the highest Bcl-xl level in the group of combined treatment compared with others, we may suggest that apoptosis was inhibited by the activation of Bcl-xl pathway. Moreover, it is known that Bcl-xl may inhibit both autophagy and apoptosis through interacting respectively with Beclin-1 and Bax. At the same time, we suggested that an autophagy in this group was triggered by the elevated levels of LC3 and Beclin-1. To summarize, we can conclude that combined therapy with metformin and PA was optimal based on the ratio of pro-apoptotic, anti-apoptotic and autophagy markers in the VMH of T2DM rats.

Despite we were trying to comprehensively characterize the processes of apoptosis and autophagy in the VMH of T2DM rats in the course of experiments, the following limitations need to be considered when interpreting the present results. Due to technical reasons, such as the small weight and size of rat VMH samples, we cannot perform the IHC studies of all proteins, therefore, we decided to only visualize protein distribution of the Bax/Bcl-xl axis. In addition, we considered caspase-3 as an apoptotic marker protein, however, it was shown that in some brain cell types caspase-3 activation plays non-apoptotic roles, including cell differentiation, cell cycle regulation, and cell migration [60]. In addition, in the CNS, caspase-3 participates in the cytoskeletal remodeling in neurons and astrocytes [61] and in the differentiation of astrocyte subpopulations [62]. The further work should be done to distinguish the non-apoptotic role of caspase-3 depending on brain cell types under the T2DM and after the treatment with metformin and PA. It is also remaining undiscovered by which mechanisms PA exerts its effects on VMH, therefore, additional investigations are further required.

In summary, our findings demonstrated the presence of the T2DM-induced activation of apoptosis and impairments in cell integrity of VMH. Diabetes-induced imbalance of pro- and anti-apoptotic proteins was more pronounced at the protein level, than at the transcriptional, indicating a significance of transcriptional homeostasis. PA administration switched apoptosis to autophagy that may mediate neuroprotective effect of PA on the background of T2DM development. Thus, based on our present findings, a combination of PA and metformin may be beneficial in modulating cell death in hypothalamus upon the development of T2DM.

## 5 Conclusions

Our data confirmed that T2DM-induced enhanced apoptosis and mitoptosis may underlie VMH nerve cell dysfunction. In turn, a tendency to slightly improve the ultrastructural changes in VMH were observed after drug administration. Metformin monotherapy led to a decrease in neuronal apoptosis, however, caused an enhanced cell aging, an accumulation of lipofuscin granules and cell debris in VMH. PA administration partially normalized VMH cytoarchitectonics and switched cell fate from apoptosis to autophagy that may be considered as adaptive and compensatory response to T2DM-induced apoptosis. Thus, PA and its combination with metformin may be effective to counteract diabetes-induced cell death by an activation of autophagy and an inhibition of apoptosis in VMH. In our opinion, PA may be considered one of the promising neuroprotective substances in addition to metformin for correcting diabetic neuropathy and impairments in hypothalamus via modulating the interplay between apoptosis and autophagy.

## Declarations

### Author contribution statement

Larysa Natrus and Nina Babel: Conceived and designed the experiments; Contributed reagents, materials, analysis tools or data.

Yuliia Osadchuk and Yuliia Klys: Performed the experiments.

Olha Lisakovska and Dmytro Labudzynskyi: Analyzed and interpreted the data; Wrote the paper.

Toralf Roch and Yuri Chaikovsky: Analyzed and interpreted the data.

### Funding statement

Nina Babel was supported by BMBF TriDiMeD [FKZ 01DK20008], NoChro [FKZ 13GW0338B].

Prof Larysa Natrus was supported by Research grant of the Ministry of Health of Ukraine [0122U001442].

### Data availability statement

Data associated with this study has been deposited at “Preprint at Research Square” under the accession number PPR449225 [DOI: 10.21203/rs.3.rs-1283211/v1].

### Declaration of interest’s statement

The authors declare no conflict of interest.

### Additional information

No additional information is available for this paper.
