# Supplementary material for: Regulation of the apoptosis/autophagy switch by propionic acid in ventromedial hypothalamus of rats with type 2 diabetes mellitus
Source: Heliyon. 2022 Nov 14;8(11):e11529. doi: 10.1016/j.heliyon.2022.e11529 (PMC9681650; doi:10.1016/j.heliyon.2022.e11529)

**Supplementary Information file 1**

**Initial set of Western blot experiments**

**(full-length, original, unprocessed versions of blots)**

**Raw data from Western blot experiments using LC3 and β-tubulin antibodies (Fig. 3a in the manuscript)**

Immunoblotting was conducted with primary antibodies against LC3 (19 kDa) and β-tubulin (55 kDa), 10 % PAAG, 50 μg of protein per track. Samples are labelled (according to the Table 1) above the images.

**Table 1 Sample labelling**

| **Gel 1** | **Gel 2** |
| --- | --- |
| 1 – marker;  2 – Ctrl (34);  3 – Ctrl (81+82);  4 – Ctrl (83 + 84);  5 – DM2 (54);  6 – DM2 (51);  7 – DM2 (52);  8 – DM2 (32);  9 – DM2 + prop. (61);  10 – DM2 + prop. (66);  11 – DM2 + prop. (62);  12 – DM2 + prop. (67);  13 – DM2 + prop. (68);  14 – DM2 + met. (43);  15 – DM2 + met. (45). | 1 – marker + DM2 (54);  2 – DM2 (51);  3 – DM2 (52);  4 – DM2 (32);  5 – DM2 + met. (41);  6 – DM2 + met. (43);  7 – DM2 + met. (45);  8 – DM2 + prop. (61);  9 – DM2 + prop. (66);  10 – DM2 + prop. (62);  11 – DM2 + prop. (67);  12 – DM2 + prop. (68);  13 – DM2 + comb. (71);  14 – DM2 + comb. (74);  15 – DM2 + comb. (75). |

**Notes:**

marker – protein ladder;

Ctrl – the control group;

DM2 – the group with experimentally induced type 2 diabetes mellitus (T2DM); DM2 + met. – the T2DM group that received anti-hyperglycemic agent metformin; DM2 + prop. – the T2DM group that received sodium salt of propionic acid (PA); DM2 + comb. – the T2DM group that received concurrently metformin and sodium salt of PA.


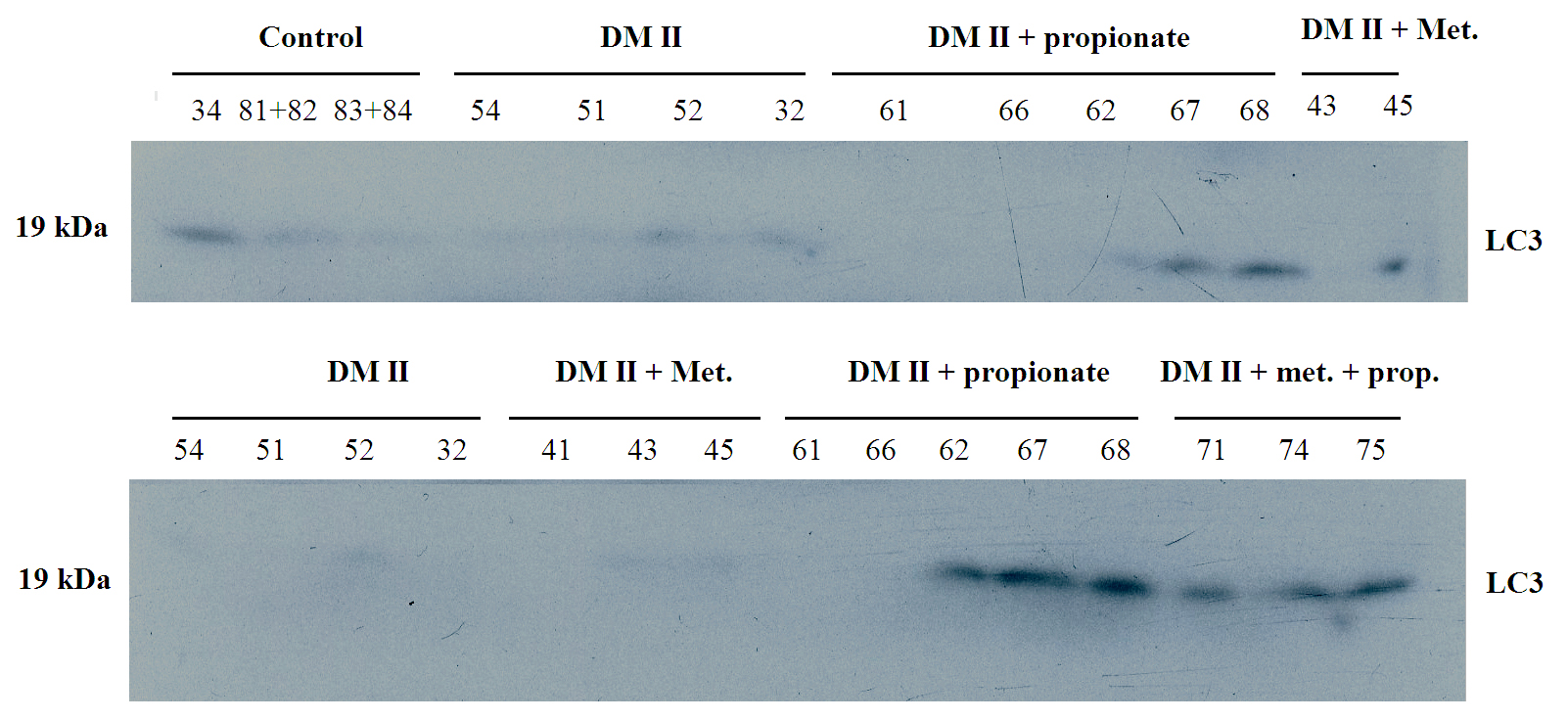


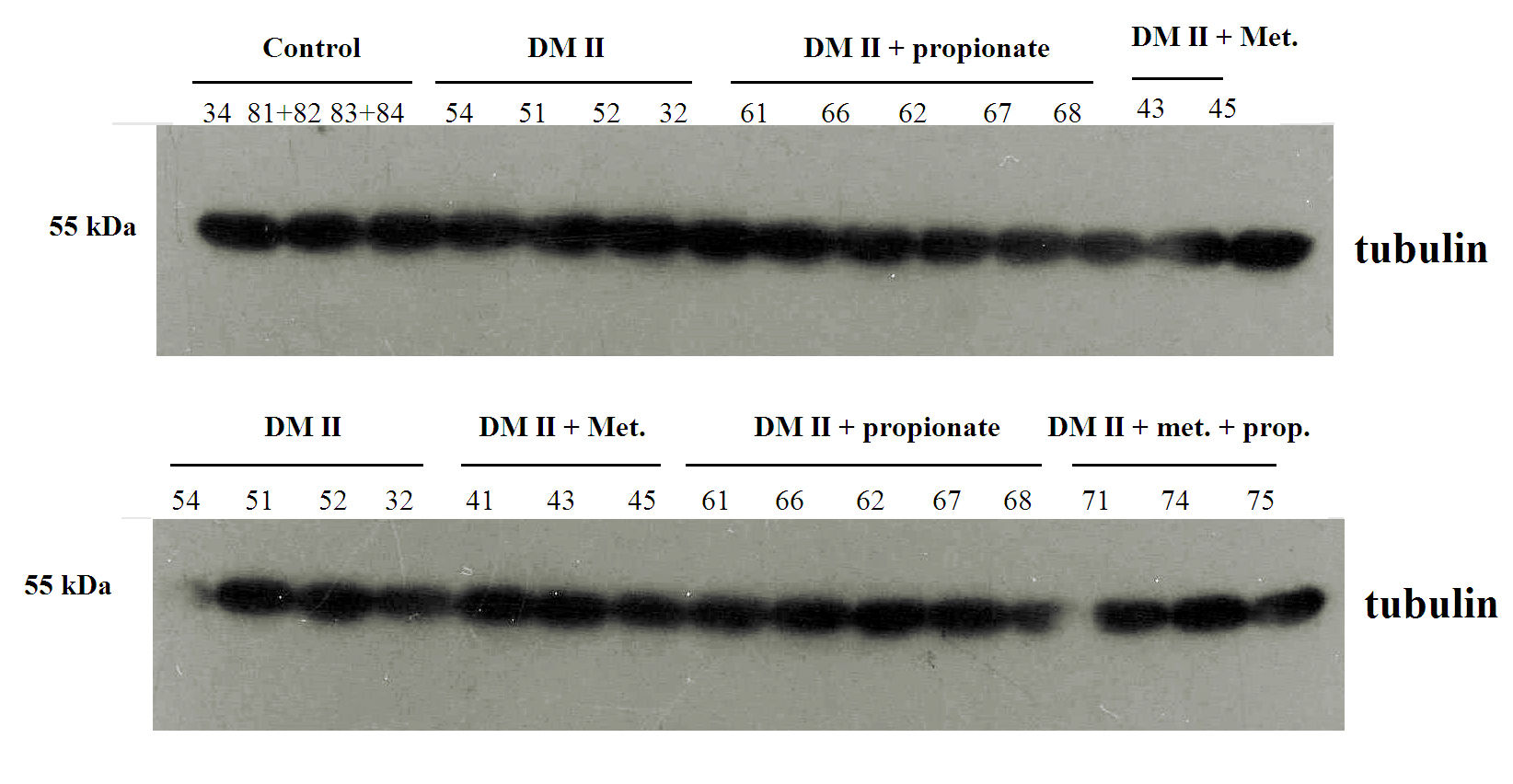


**Raw data from Western blot experiments using Beclin-1 (Fig. 3a in the manuscript) and Bcl-xL (Fig. 3f in the manuscript) antibodies**

Immunoblotting was conducted with primary antibodies against Beclin-1 (52 kDa) and Bcl-xL (26 kDa), 8 % PAAG, 50 μg of protein per track. Samples are labelled (according to the Table 2) above the images.

**Table 2 Sample labelling**

| **Gel 1** | **Gel 2** |
| --- | --- |
| 1 – marker;  2 – Ctrl (32);  3 – Ctrl (34);  4 – Ctrl (81+82);  5 – Ctrl (83 + 84);  6 – DM2 (54);  7 – DM2 (51);  8 – DM2 (52);  9 – DM2 + prop. (61);  10 – DM2 + prop. (66);  11 – DM2 + prop. (62);  12 – DM2 + prop. (67);  13 – DM2 + prop. (68);  14 – DM2 + met. (43);  15 – DM2 + met. (45). | 1 – marker;  2 – DM2 (54);  3 – DM2 (51);  4 – DM2 (52);  5 – DM2 + met. (41);  6 – DM2 + met. (43);  7 – DM2 + met. (45);  8 – DM2 + prop. (61);  9 – DM2 + prop. (66);  10 – DM2 + prop. (62);  11 – DM2 + prop. (67);  12 – DM2 + prop. (68);  13 – DM2 + comb. (71);  14 – DM2 + comb. (74);  15 – DM2 + comb. (75). |

**Notes:**

marker – protein ladder;

Ctrl – the control group;

DM2 – the group with experimentally induced type 2 diabetes mellitus (T2DM); DM2 + met. – the T2DM group that received anti-hyperglycemic agent metformin; DM2 + prop. – the T2DM group that received sodium salt of propionic acid (PA); DM2 + comb. – the T2DM group that received concurrently metformin and sodium salt of PA.


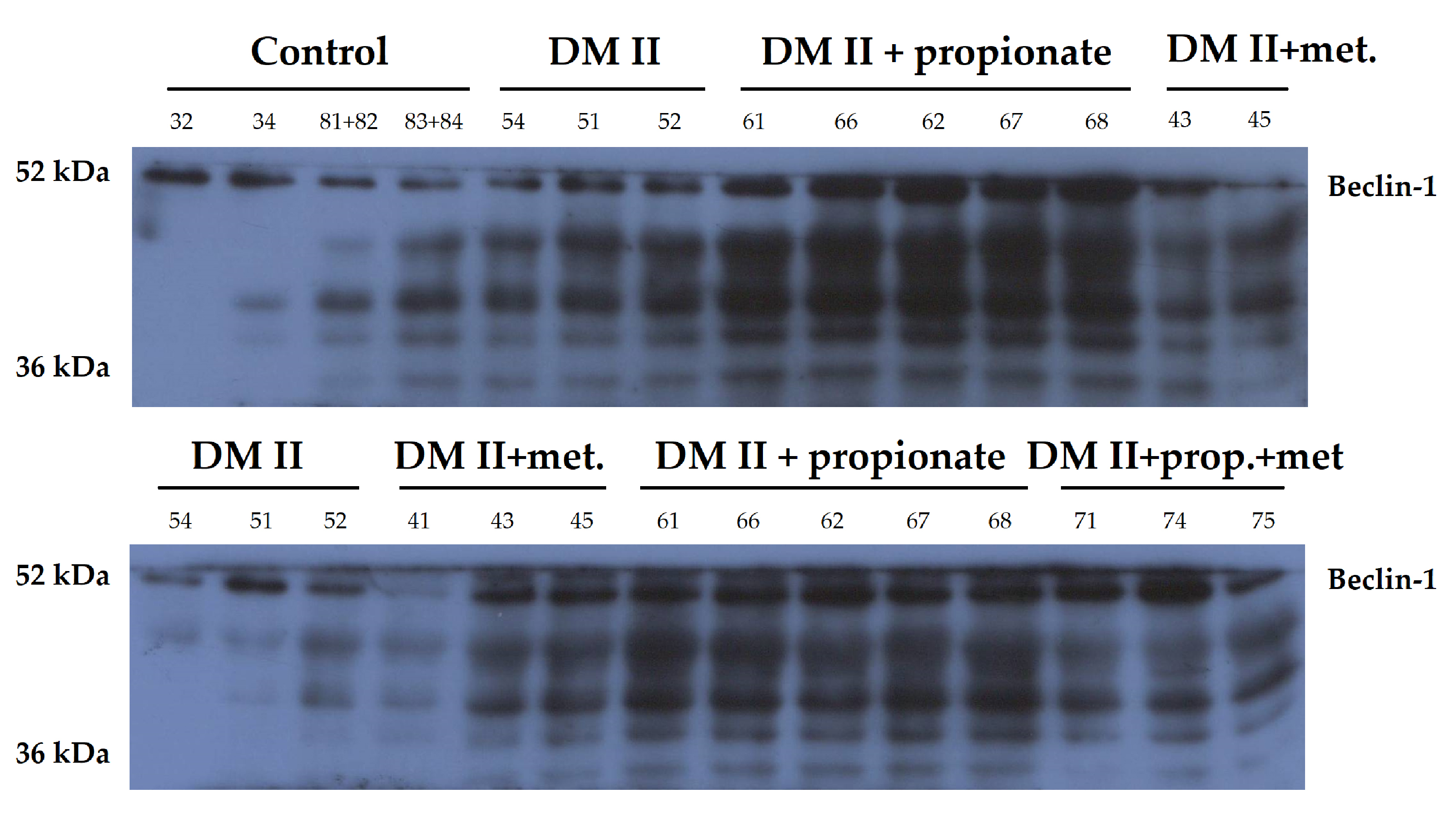


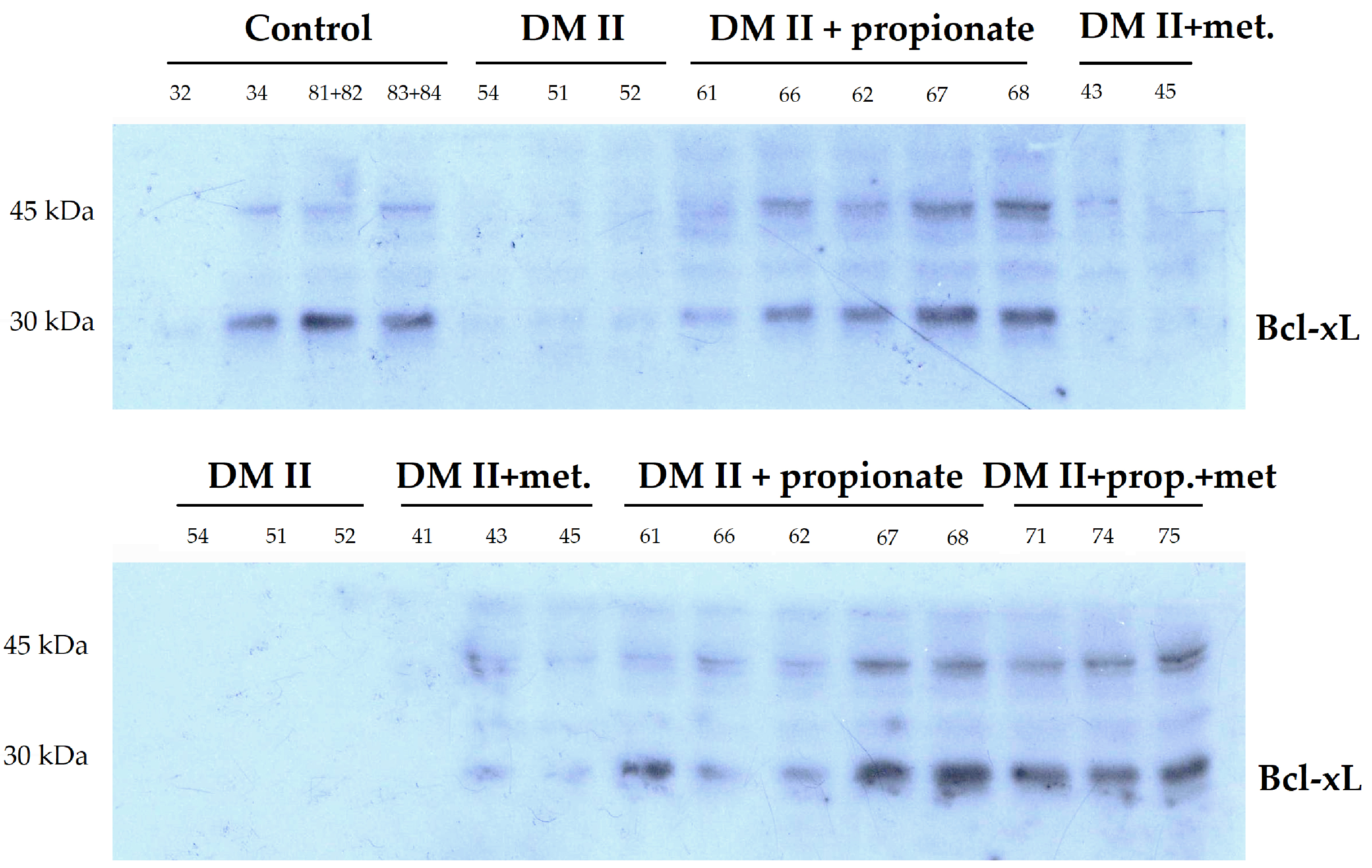

Supplement: Supplementary_Material_1 [file mmc1.doc]
